# Supplementary material for: Transformation of dolutegravir into an ultra-long-acting parenteral prodrug formulation
Source: Nat Commun. 2022 Jun 9;13:3226. doi: 10.1038/s41467-022-30902-7 (PMC9184486; doi:10.1038/s41467-022-30902-7)
Supplement: Supplementary file 1 — Supplementary Information [file 41467_2022_30902_MOESM1_ESM.pdf]

Supplementary Information: Transformation of Dolutegravir into an Ultra-Long-Acting Parenteral Prodrug Formulation

H.E. Gendelman et al.

This file includes:

Supplementary Methods

Supplementary Fig. 1-19

Supplementary Table 1-5

Supplementary Methods

DTG prodrugs chemical characterization

Proton (^{1}H)- and carbon (^{13}C)- NMR spectra for MDTG, M2DTG, M3DTG, and M4DTG were recorded on a Varian Unity/Inova-500 NB (500 MHz, Varian Medical Systems Inc., Palo Alto, CA)

MDTG: ^{1}H NMR (500 MHz, $CDCl_3$): δ 10.20 (s, 1 H), 8.45 (s, 1 H), 7.35 (dd, J = 15.0, 8.2 Hz, 1 H), 6.83 (app. dd, J = 19.1, 9.3 Hz, 1 H), 5.26 (br. s, 1 H), 4.85–5.01 (m, 1 H), 4.62 (br. s, 1 H), 4.30 (app. d, J = 12 Hz, 2 H), 4.17 (dd, J = 13.3, 5.9 Hz 1 H), 4.0 (app. d, J = 6.3 Hz, 1 H), 2.73

(t, J = 7.6 Hz, 2 H), 2.17 (td, J = 14.5, 7.2 Hz, 1 H), 1.80 (app. t, J = 7.5 Hz, 2 H), 1.51–1.61 (m, 2 H), 1.40–1.49 (m, 2 H), 1.36 (d, J = 7.0 Hz, 3 H), 1.26 (br. s, 20 H), 0.90 (t, J = 6.6 Hz, 3 H).

^{13}C NMR (125 MHz, $CDCl_3$): δ 171.9, 171.1, 163.3, 163.1, 161.6, 161.1, 159.6, 130.6, 130.5, 129.2, 121.3, 121.2, 119.5, 111.2, 111.1, 103.9, 103.7, 103.5, 76.1, 53.1, 36.5, 33.8, 31.9, 29.7, 29.6, 29.5, 29.4, 29.3, 29.2, 29.0, 24.4, 22.6, 15.8, 14.1.

HRMS (ESI) m/z: $[M + H]^+$: calculated for $C_{34}H_{45}F_2N_3O_6$, 629.33 (100%), 630.33 (36.8%), 631.33 (3.9%); found, 630.30.

M2DTG: ^1H NMR (500 MHz, $CDCl_3$): 10.18 (t, J = 5.7 Hz, 1H), 8.44 (s, 1H), 7.33 (dd, J = 14.9, 8.3 Hz, 1H), 6.75-6.85 (m, 2H), 5.23 (t, J = 4.6 Hz, 1H), 4.94 (t, J = 6.1 Hz, 1H), 4.60 (b, 2H), 4.29 (app. d, J = 10.4 Hz, 1H), 4.16 (dd, J = 13.4, 6.0 Hz 1H), 2.71 (t, J = 7.7 Hz, 2H), 1.78 (p, J = 7.7 Hz, 2H), 1.42 (p, J = 7.3 Hz, 2H), 1.34 (d, J = 7.0 Hz, 3H), 1.25 (b, 28H), 0.88 (t, J = 6.9 Hz, 3H).

^{13}C NMR (125 MHz, $CDCl_3$): δ 172.0, 171.1, 163.4, 163.3, 163.2, 161.8, 161.3, 159.8, 143.1, 130.7, 130.6, 130.5, 129.2, 121.3, 121.2, 119.6, 111.3, 111.1, 103.9, 103.8, 103.6, 76.2, 53.1, 36.6, 33.9, 31.9, 29.7, 29.6, 29.5, 29.3, 29.2, 29.1, 24.5, 22.7, 15.9, 14.1.

HRMS (ESI) m/z: $[M + H]^+$: calculated for $C_{38}H_{54}F_2N_3O_6^+$, 685.39 (100%), 686.39 (41.1%), 687.40 (8.2%); found, 686.38.

M3DTG: ^1H NMR (500 MHz, $CDCl_3$): 10.17 (t, J = 5.7 Hz, 1H), 8.42 (s, 1H), 7.33 (dd, J = 14.9, 8.4 Hz, 1H), 6.75-6.85 (m, 2H), 5.23 (t, J = 4.8 Hz, 1H), 4.95 (t, J = 6.1 Hz, 1H), 4.60 (b, 2H), 4.28

(d, J = 12.2 Hz, 1H), 4.15 (dd, J = 13.4, 6.0 Hz 1H), 3.98 (d, J = 7.2 Hz, 2H), 2.71 (t, J = 7.7 Hz, 2H), 2.18 (tt, J = 14.5, 7.2 Hz, 2H), 1.78 (p, J = 7.6 Hz, 2H), 1.42 (p, J = 7.3 Hz, 2H), 1.33 (d, J = 7.0 Hz, 3H), 1.25 (b, 36H), 0.88 (t, J = 6.9 Hz, 3H).

^{13}C NMR (125 MHz, $CDCl_3$): δ 172.0, 171.1, 163.4, 163.3, 163.2, 161.7, 161.2, 159.7, 143.2, 130.7, 130.6, 130.5, 129.2, 121.3, 121.2, 119.6, 111.3, 111.1, 103.9, 103.8, 103.6, 76.2, 53.1, 36.5, 33.9, 31.9, 29.7, 29.6, 29.5, 29.3, 29.2, 29.1, 24.5, 22.7, 15.9, 14.1.

HRMS (ESI) m/z: $[M + H]^+$: calculated for $C_{42}H_{62}F_2N_3O_6^+$, 741.45 (100%), 742.46 (45.4%), 743.46 (7.4%); found, 742.40.

M4DTG: ^{1}H NMR (500 MHz, $CDCl_3$): 10.21 (t, J = 5.7 Hz, 2H), 8.50 (s, 2H), 7.33 (dd, J = 14.9, 8.3 Hz, 2H), 6.75-6.85 (m, 4H), 5.21 (t, J = 3.9 Hz, 2H), 4.92 (t, J = 6.0 Hz, 2H), 4.59 (b, 4H), 4.33 (dd, J = 13.4, 3.8 Hz, 2H), 4.18 (dd, J = 12.7, 4.8 Hz 2H), 3.96 (d, J = 6.7 Hz, 4H), 2.71 (t, J = 7.7 Hz, 4H), 2.17 (tt, J = 14.5, 7.2 Hz, 2H), 1.72-1.84 (m, 5H), 1.42 (p, J = 7.2 Hz, 4H), 1.32 (d, J = 7.0 Hz, 6H), 1.25 (b, 24H).

^{13}C NMR (125 MHz, $CDCl_3$): δ 172.0, 171.1, 163.4, 163.3, 163.2, 161.7, 161.2, 159.7, 143.2, 130.7, 130.6, 130.5, 129.2, 121.3, 121.2, 119.6, 111.3, 111.1, 103.9, 103.8, 103.6, 76.2, 53.1, 36.6, 33.7, 29.7, 29.6, 29.5, 29.3, 29.1, 24.5, 15.9.

HRMS (ESI) m/z: $[M + H]^+$: calculated for $C_{58}H_{69}F_4N_6O_{12}^+$, 1116.48 (100%), 1117.49 (62.7%), 1118.49 (19.3%); found, 1117.45.

Thermal analyses

Differential scanning calorimetry (DSC) thermograms were obtained using a Discovery X3 Differential Scanning Calorimeter (TA Instruments, New Castle, DE, USA), while thermogravimetric analysis (TGA) was performed using a TGA 550 (TA Instruments, New Castle, DE, USA) with a thermal analysis operating system (TA DSC 25) and TRIOS software V5.1.1 (Waters/TA Instruments, New Castle, DE, USA). For DSC, about 8 mg of sample was hermetically sealed in an aluminum pan (Tzero pans, TA Instruments), while an empty pan was used as a reference. The pans were heated from 30 - 300 °C at the rate of 10 °C/min in a nitrogen environment (flow rate 20 mL/min). TGA was performed from 30 - 300 °C at the rate of 10 °C/min in a nitrogen environment (flow rate 20 mL/min).

Cytotoxicity assays

Following treatment with free drug and nanoparticles, cell vitality of MDMs was evaluated using the 3-(4,5-dimethylthiazol-2-yl)-2,5-diphenyltetrazolium bromide (MTT) assay as detailed in our previous works (29, 30). The treatments comprised of NDTG or NM2DTG from 1.95 – 1,000 µM or 2-fold dilutions of DTG or M2DTG from 0.20 - 100 µM for 24 h. Untreated and vehicle-treated cells were used as controls. For each group, quadruplicate samples were used. The absorbance was measured at 490 nm on a Molecular Devices SpectraMax M3 plate reader with SoftMax Pro 6.2 software (Sunnyvale, CA, USA). Absorbance was compared to that of untreated control cells to determine cell vitality.

UPLC-TUV quantification of DTG and prodrugs

A Waters ACQUITY UPLC H-Class system with tunable ultraviolet/visible (TUV) detector and Empower 3 software measured drug concentrations. DTG, MDTG, M2DTG, and M3DTG samples were separated on a Phenomenex Kinetex 5 µm C18 column (150 × 4.6 mm) (Torrance, CA, USA). DTG was detected at 254 nm, using isocratic elution with a mobile phase consisting of 65% 50 mM KH_2PO_4, pH 3.2/35% ACN at a flow rate of 1.0 mL/min (29). MDTG, M2DTG, and M3DTG were detected at 230 nm, using isocratic elution with mobile phases consisting of 90% ACN/10% water, 95% ACN/5% water, or 98% ACN/2% water ratio at a flow rate of 1.0 mL/min. M4DTG samples were separated on a Waters XBridge 3.5 µm C8 column (150 × 3.0 mm) (Milford, MA, USA). M4DTG was detected at 230 nm, using isocratic elution with a mobile phase consisting of 80% ACN/20% water ratio and 0.5 mL/min flow rate. Drug content was determined relative to peak areas of drug standards (0.05–50 µg/mL) in methanol.

Quantitation of DTG and M2DTG by UPLC-MS/MS

DTG and M2DTG were quantitated in mouse, rat, and rhesus macaque plasma and tissues by UPLC-MS/MS using a Waters ACQUITY H-class UPLC (Milford, MA, USA) connected to a Xevo TQ-S micro mass spectrometer and analyzed using Waters MassLynx V4.2 software (Milford, MA, USA). All solvents for sample processing and UPLC-MS/MS analysis were LC-MS-grade (Fisher). For rodent plasma and blood samples, 25 µL of sample was added into 1 mL ACN spiked with 10 µL internal standard (IS). Samples were vortexed and centrifuged at 17,000

× g for 10 minutes at 4 °C. The supernatants were collected and dried using a SpeedVac and reconstituted in 100 µL 20% water/80% methanol; 10 µL was injected for DTG, and M2DTG UPLC–MS/MS analyses. Standard curves were prepared in blank mouse or rhesus macaque plasma/blood in the range of 0.2-1000 ng/mL for respective analytes. For rodent tissue drug quantitation, 3-200 mg of sample was homogenized in 4-29 volumes of 0.1% v/v formic acid and 2.5 mM ammonium formate containing 90% methanol. 280 µL of Optima grade methanol containing 0.1% formic acid and 2.5 mM ammonium formate was added to 100 µL of rodent tissue homogenate, followed by addition of 80% methanol (10 µL), and IS (10 µL), followed by vortexing for 3 minutes and centrifugation at 20,000 × g for 15 minutes. d3-Dolutegravir (d3-DTG), myristoylated darunavir (MDRV) and stearoylated darunavir (SDRV), at a final concentration of 40, 20 and 20 ng/mL, respectively, were used as ISs for DTG and M2DTG analyses respectively. Finally, 85 µl of supernatant was mixed with 15 µL Optima grade water, and 10 µL was injected for DTG, and M2DTG UPLC–MS/MS analyses. Standards were prepared similarly using blank tissue homogenates with 10 µL of spiking solution (DTG/M2DTG, 5-5,000 ng/mL in 80% MeOH containing 0.1% formic acid and 2.5 mM ammonium formate). For DTG quantitation, chromatographic separation of 10 µL DTG sample was performed on a Waters ACQUITY UPLC BEH Shield RP18 column (1.7 µm, 2.1 mm x 100 mm) using a 10-minute gradient of mobile phase A (7.5 mM ammonium formate in water, adjusted to pH 3 using formic acid) and mobile phase B (100% ACN) at a flow rate of 0.25 mL/minute. For the first 3.5 minutes, the mobile phase composition was 35% B and was increased to 95% B in 0.5 minute and held constant for 1.5 minutes. Mobile phase B was then reset to 35% in 0.5 minute, and the column was

equilibrated for 1 minute before the next injection. Chromatographic separation for M2DTG quantitation was achieved on a Waters ACQUITY UPLC BEH Shield RP18 column (1.7 µm, 2.1 mm × 30 mm) using an 8-minute gradient of mobile phase A (7.5 mM ammonium formate in water, adjusted to pH 3 using formic acid) and mobile phase B (100% methanol) at a flow rate of 0.28 mL/minute. For M2DTG, the initial mobile phase composition was 85% B for the first 5 minutes, and increased to 95% B in 0.25 minute, held constant for 1 minute, reset to 85% in 0.25 minute and the column was equilibrated for 1 minute before the next injection. DTG and M2DTG were detected at a cone voltage of 10 V and 8 V, respectively, and a collision energy of 25 V and 30 V, and 50 V, 44 V, 14V, respectively. Multiple reaction monitoring (MRM) transitions used for DTG, M2DTG, d3-DTG, MDRV, and SDRV were 420.07>127.04, 420.07>277.12, 420.07>295.12, 686.38>126.96, 686.38>277.02, 686.38>420.07, 758.64> 602.55, 814.457>658.403, respectively. Spectra were analyzed and quantified by Waters MassLynx V4.2 software (Milford, MA, USA). All quantitation's were determined using analyte peak area to internal standard peak area ratios.

Carboxylesterase quantification

ELISA kits for the quantification of specific carboxylesterases for Rat Carboxylesterase 1 (CES1; catalog no. MBS452470) and Rat Cocaine esterase (CES2; catalog no. MBS7216469) were purchased from MyBioSource (San Diego, CA, USA) and used per manufacturer instructions. Male SD rats (SASCO) were euthanized using isoflurane, exsanguinated, and subjected to whole body perfusion. Tissues, including liver, kidney, muscle, spleen, lymph nodes, and gut were

collected and homogenized according to protocols established previously (29, 30) in 5 volumes of ice-cold PBS. Rat plasma was used without homogenization or dilution. Heat-inactivation was achieved by incubation at 60 °C for a minimum of 20 min. All the kit components and the samples were brought to room temperature before use and processed as per the protocols provided with the kits. The plates were analyzed at 450 nm on a Molecular Devices SpectraMax M3 plate reader with SoftMax Pro 6.2 software (Sunnyvale, CA, USA). CES concentrations were calculated relative to the absorbance of the standard curve after normalizing to blanks.

Molecular docking

A series of induced fit molecular docking simulations (Maestro Suite 2020-2, Schrödinger, New York City, NY, USA) were conducted with Carboxylesterase 1. The three-dimensional crystal structures of CES1 (PDB ID: 1YA8; PDB DOI: 10.2210/pdb1YA8/pdb) protein were retrieved from the Research Collaboratory for Structural Bioinformatics Protein Data Bank (RCBS PDB) database. Structure optimization was performed using the Protein Preparation Wizard (Epik, Impact, and Prime). Prodrug structures were prepared prior to docking using the LigPrep application for the conversion of structures from 2-dimensional to 3-dimensional, correction of improper bond distances, bond orders, generation of ionization states, and energy minimization processes. The prepared protein and ligand structures were then used for Induced Fit Docking (IFD). Flexible protein-ligand docking was performed using the Induced Fit Docking application, which combines Grid-based Ligand Docking with Energetics (GLIDE) and Prime refinement modules. The ligand 2-methylbutanoic acid in the crystal 1YA8 binding site complex was used to

define the binding site. It was then used as the centroid of the workplace, which represents protein putative functional sites. SiteMap module was used to highlight regions within the binding site suitable for occupancy by hydrophobic groups or by ligand hydrogen-bond donors, acceptors, or metal-binding.

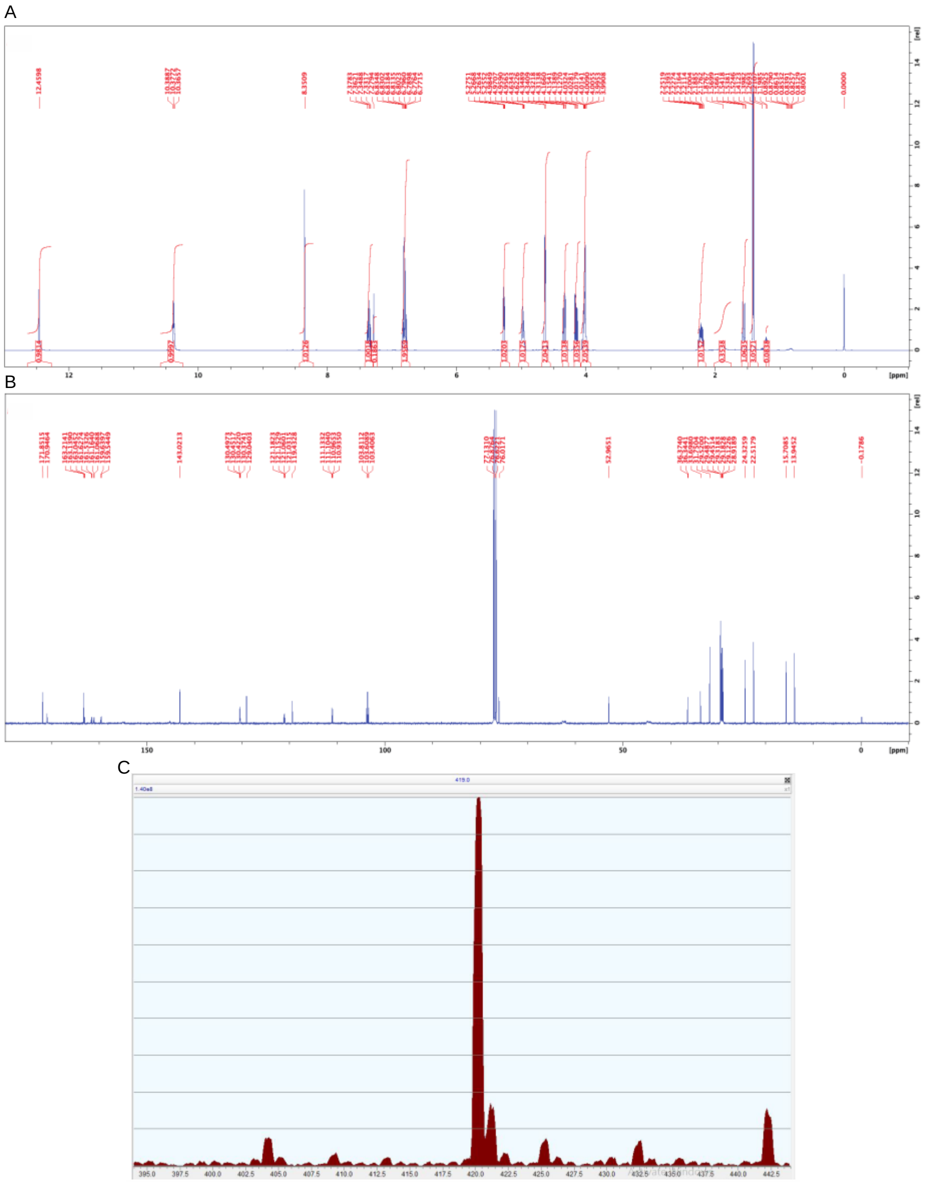

Supplementary Fig. 1. Structural characterization of DTG. (A) ^{1}H and (B) ^{13}C NMR of DTG. (C) ESI infusion of DTG generated a signal at 419.904 m/z. (A-C) Experiments were repeated five independent times with equivalent results.

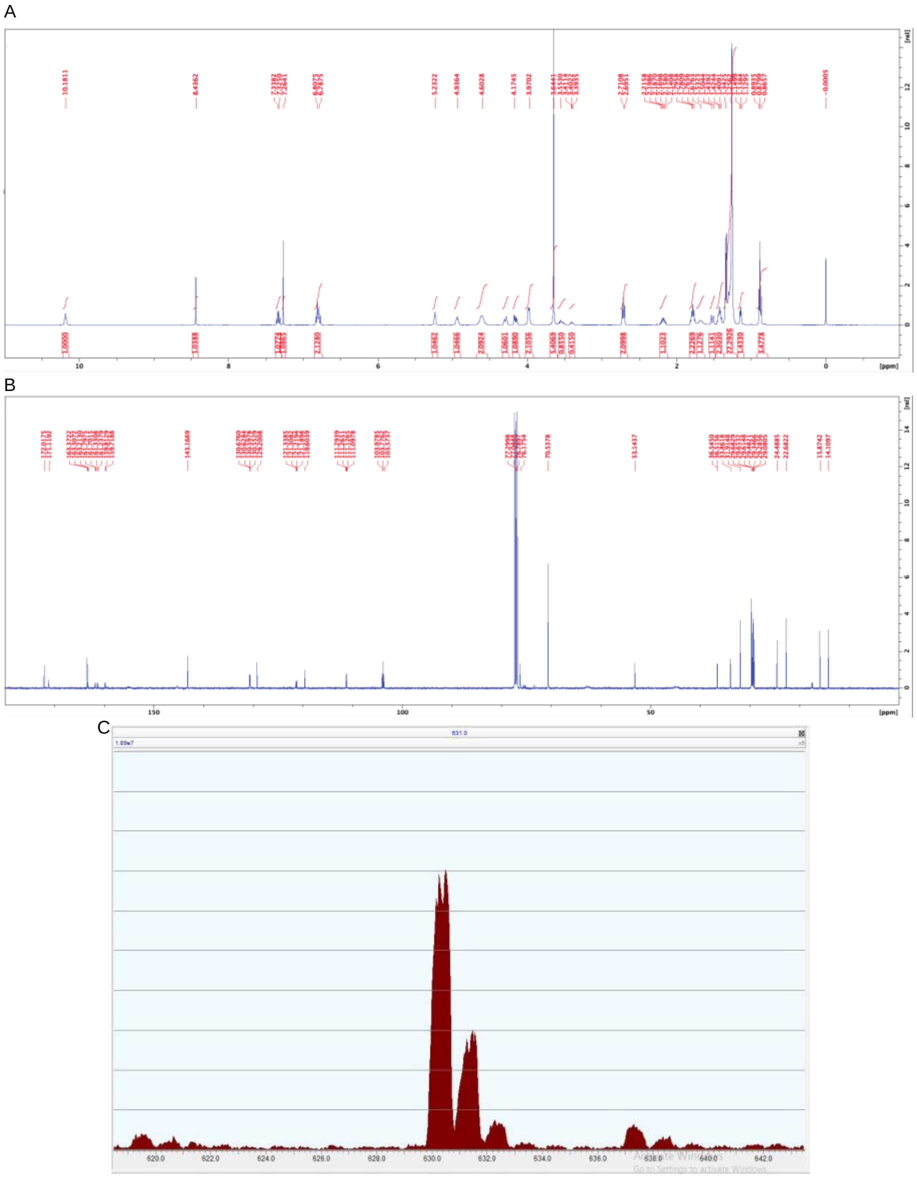

Supplementary Fig. 2. Structural characterization of MDTG. (A) ^{1}H and (B) ^{13}C NMR of MDTG. (C) ESI infusion of MDTG generated a strong signal at 630.3 m/z. (A-C) Experiments were repeated five independent times with equivalent results.

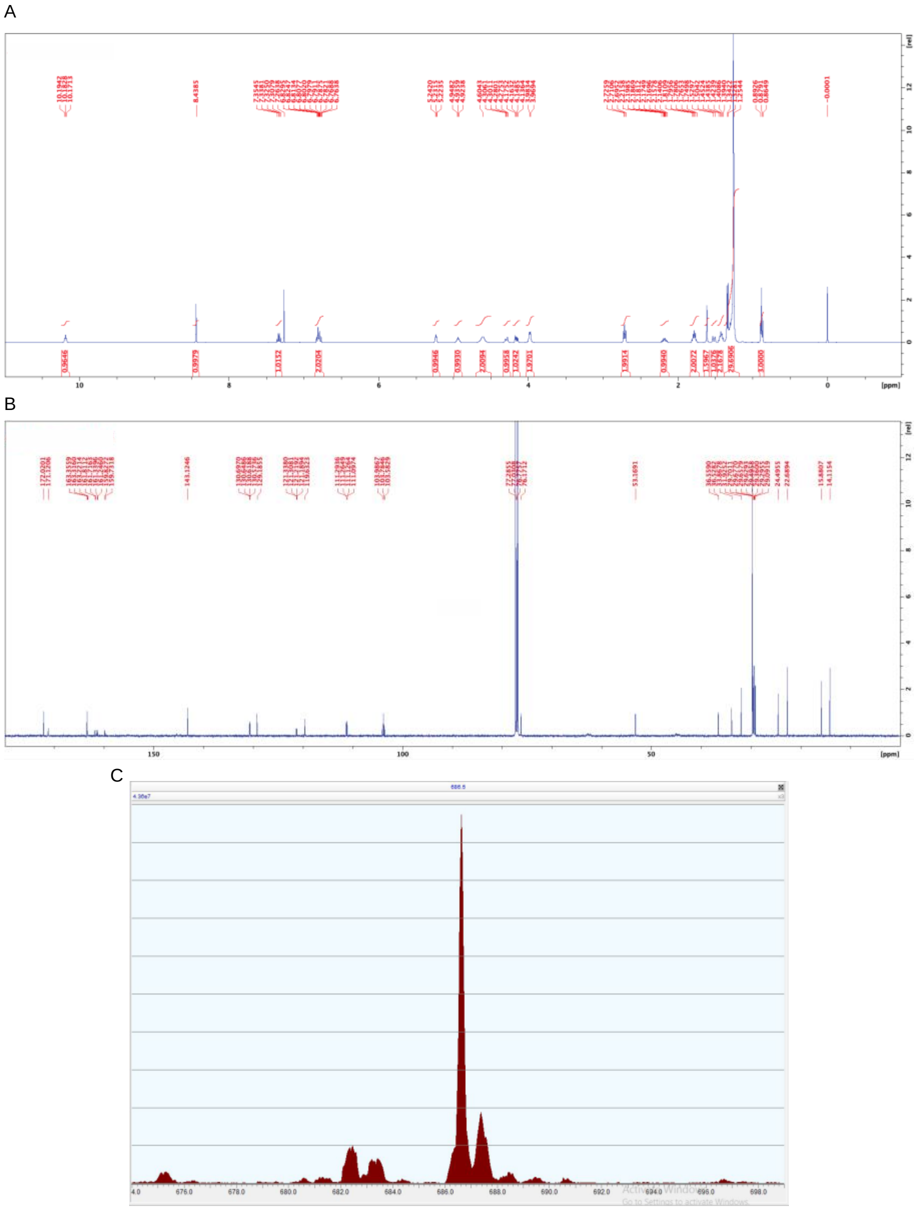

Supplementary Fig. 3. Structural characterization of M2DTG. (A) ^{1}H and (B) ^{13}C NMR of M2DTG. (C) ESI infusion of M2DTG generated a strong signal at 686.38 m/z. (A-C) Experiments were repeated five independent times with equivalent results.

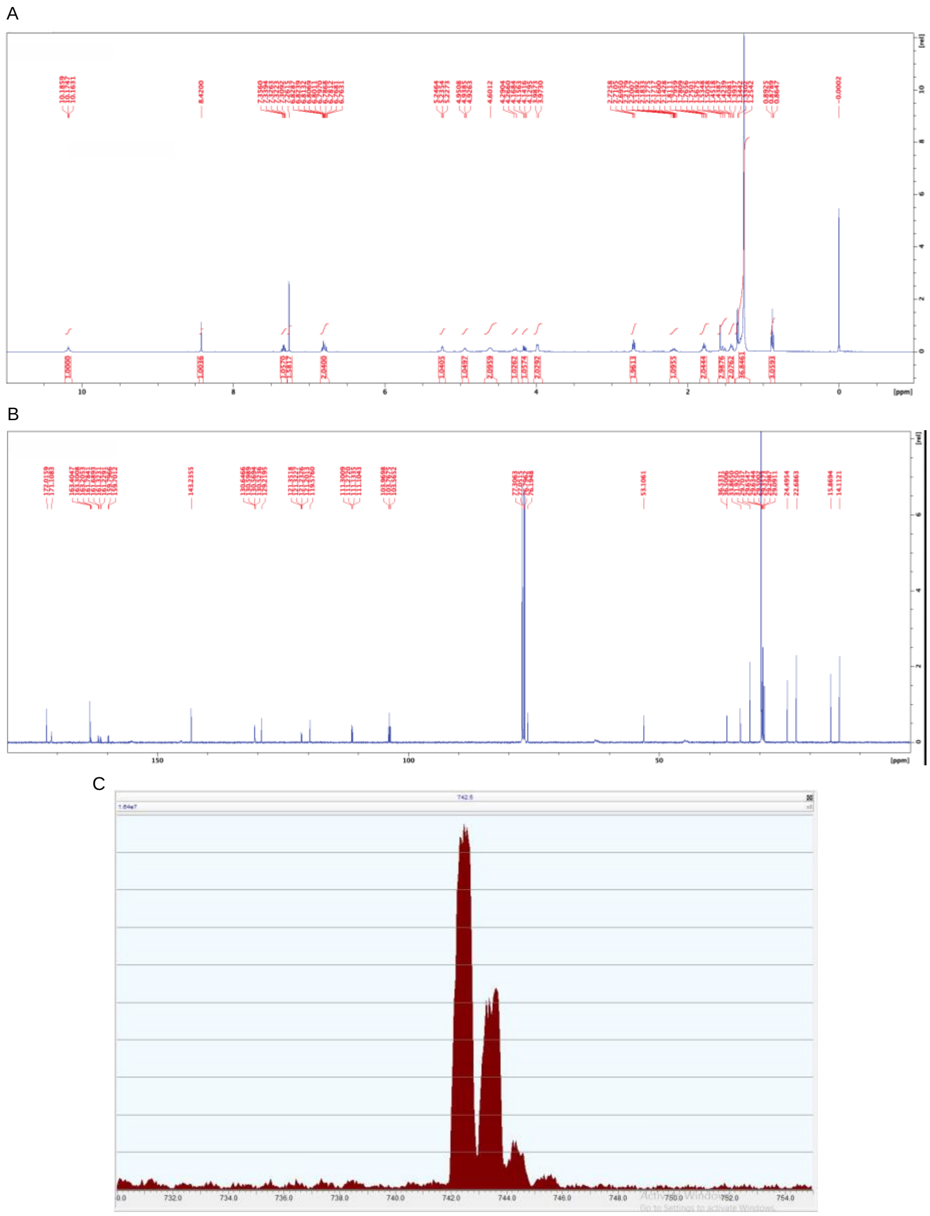

Supplementary Fig. 4. Structural characterization of M3DTG. (A) ^{1}H and (B) ^{13}C NMR of M3DTG. (C) ESI infusion of M3DTG generated a strong signal at 742.4 m/z. (A-C) Experiments were repeated five independent times with equivalent results.

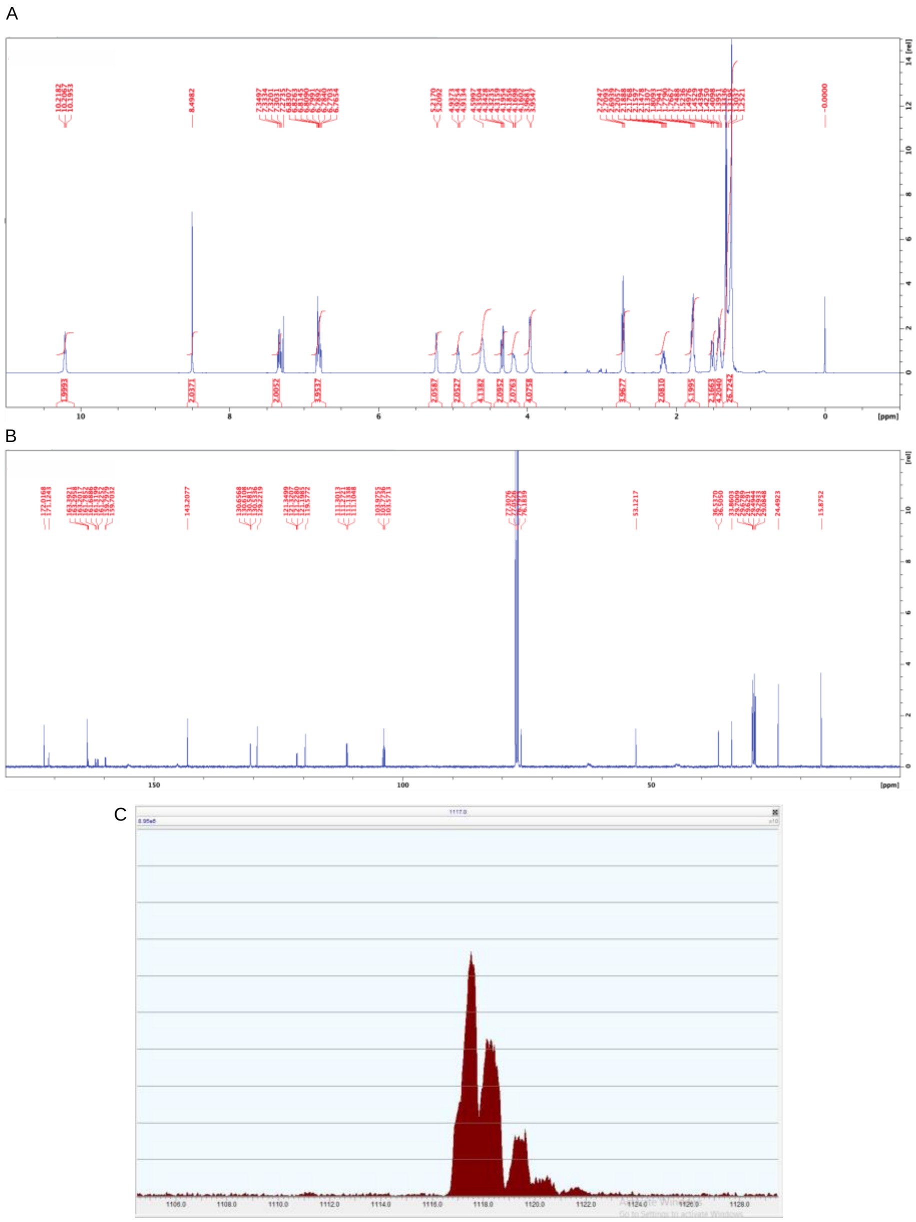

Supplementary Fig. 5. Structural characterization of M4DTG. (A) ^{1}H and (B) ^{13}C NMR of M4DTG. (C) ESI infusion of M4DTG generated a strong signal at 1,117.45 m/z. (A-C) Experiments were repeated five independent times with equivalent results.

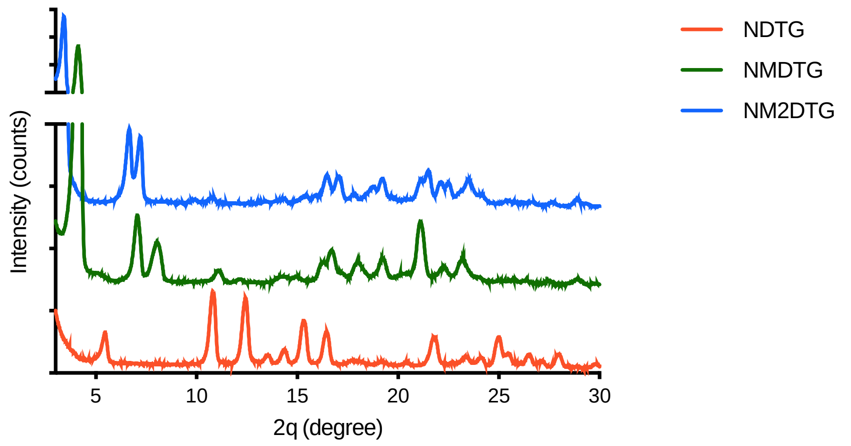

Supplementary Fig. 6. X-ray diffraction (XRD) of nanoformulated DTG and prodrugs. Overlay of powder X-ray diffraction (XRD) patterns of lyophilized NDTG (orange), NMDTG (green), and NM2DTG (blue) nanoformulations at 2θ = 2-70 at 1°/min. Source data are provided in the Figshare database under Digital Object Identifier (DOI) code 10.6084/m9.figshare.19027397.

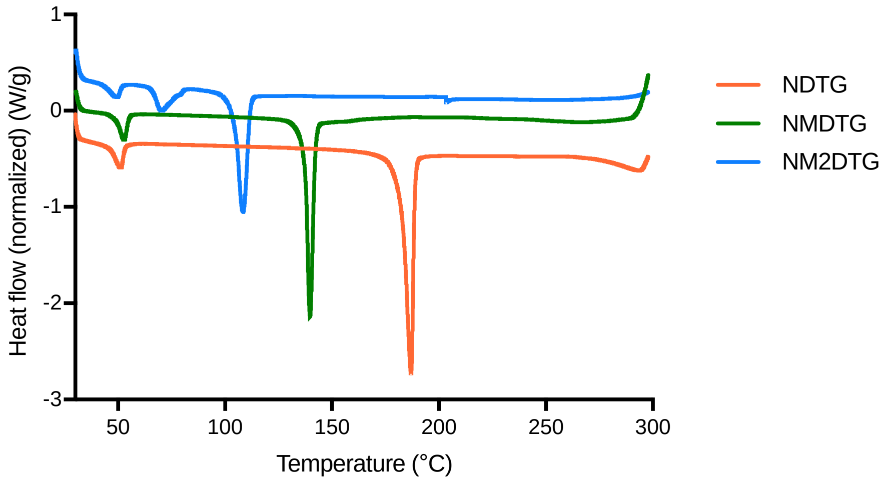

Supplementary Fig. 7. Differential scanning calorimetry of DTG prodrug nanoformulations. Overlay of differential scanning calorimetry (DSC) thermograms of lyophilized NDTG (orange), NMDTG (green), and NM2DTG (blue) nanoformulations at a ramp of 10 °C/min from 30-300 °C. Source data are provided in the Figshare database under Digital Object Identifier (DOI) code 10.6084/m9.figshare.19027397.

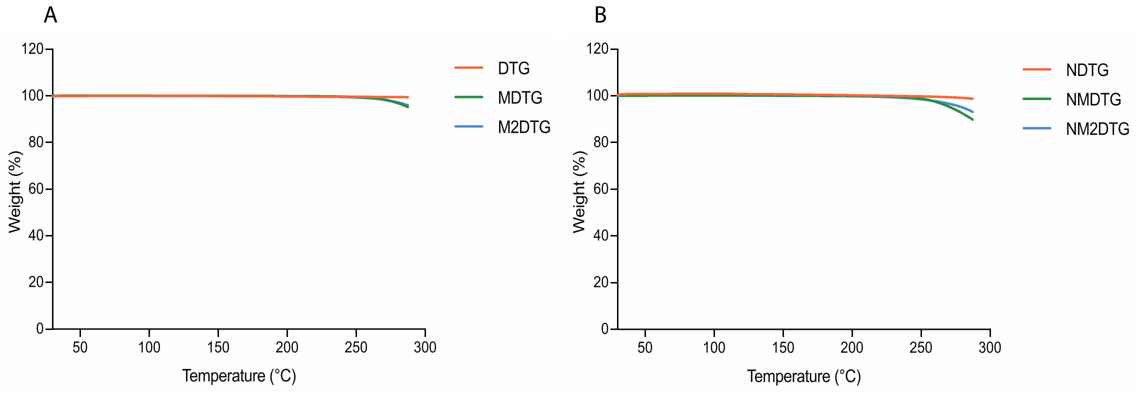

Supplementary Fig. 8. Thermogravimetric analyses (TGA) of DTG, prodrugs, and nanoformulations. (A) Overlay of TGA thermograms of DTG (orange), MDTG (green), and M2DTG (blue) at a ramp of 10 °C/min from 30-300 °C. (B) Overlay of TGA thermograms of lyophilized NDTG (orange), NMDTG (green), and NM2DTG (blue) nanoformulations at a ramp of 10 °C/min from 30-300 °C. Source data are provided in the Figshare database under Digital Object Identifier (DOI) code 10.6084/m9.figshare.19027397.

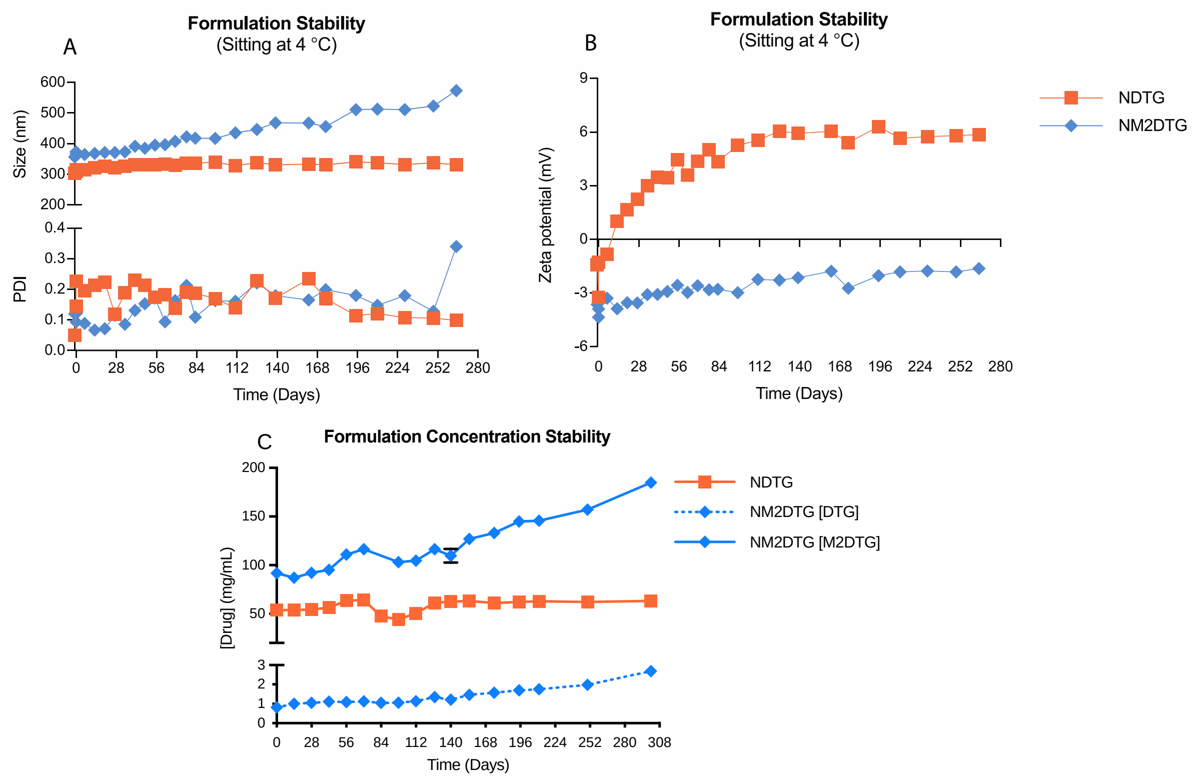

Supplementary Fig. 9. Nanoformulation stability. (A) Hydrodynamic diameter (size), polydispersity index (PDI), and **(B)** zeta potential of NDTG (orange) and NM2DTG (blue) nanoformulations at 4 °C were evaluated over 265 days, as determined by dynamic light scattering (DLS). **(C)** Drug concentration stability of NDTG (orange) and NM2DTG (blue; [M2DTG] – solid line, [DTG] – dotted line) within the nanoformulation. Results are expressed as the mean ± SEM for N = 3 independent replicates. Replicate concentration samples were each quantified in duplicate. Source data are provided in the Figshare database under Digital Object Identifier (DOI) code 10.6084/m9.figshare.19027397.

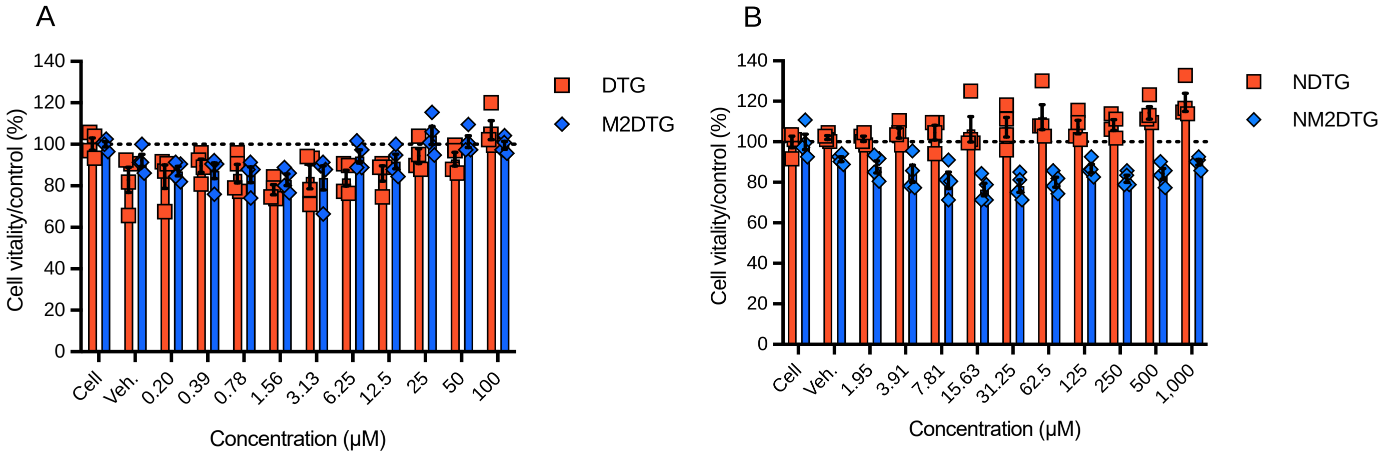

Supplementary Fig. 10. Cytotoxicity assay. Cell vitality was assessed in MDM by MTT assay after 24 h treatment with (**A**) DTG (orange) or M2DTG (blue) over a range of concentrations (0.2 - 100 μM); or (**B**) NDTG (orange) or NM2DTG (blue) nanoformulations over a range of concentrations (1.95 - 1000 μM). Results were normalized to untreated control cells. Results are expressed as the mean ± SEM for N = 4 biological replicates. Source data are provided in the Figshare database under Digital Object Identifier (DOI) code 10.6084/m9.figshare.19027397.

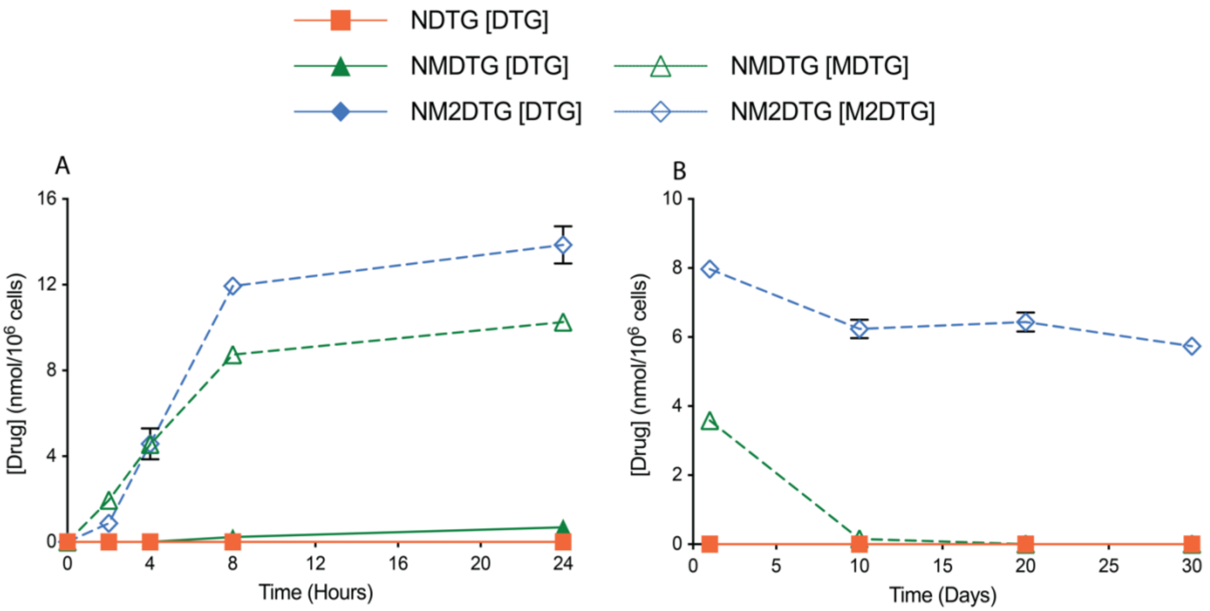

Supplementary Fig. 11. Pharmacological characterization of NM2DTG in human monocyte-derived macrophages. (**A**) Drug uptake in MDM was measured over a 24-h period after treatment with drug concentration of 5 μM. (B) Drug retention in MDM was measured over a 30-day observation period after treatment with drug concentration of 5 μM for 8 h. Results are expressed as the mean ± SEM for N = 3 biological replicates. Source data are provided in the Figshare database under Digital Object Identifier (DOI) code 10.6084/m9.figshare.19027397.

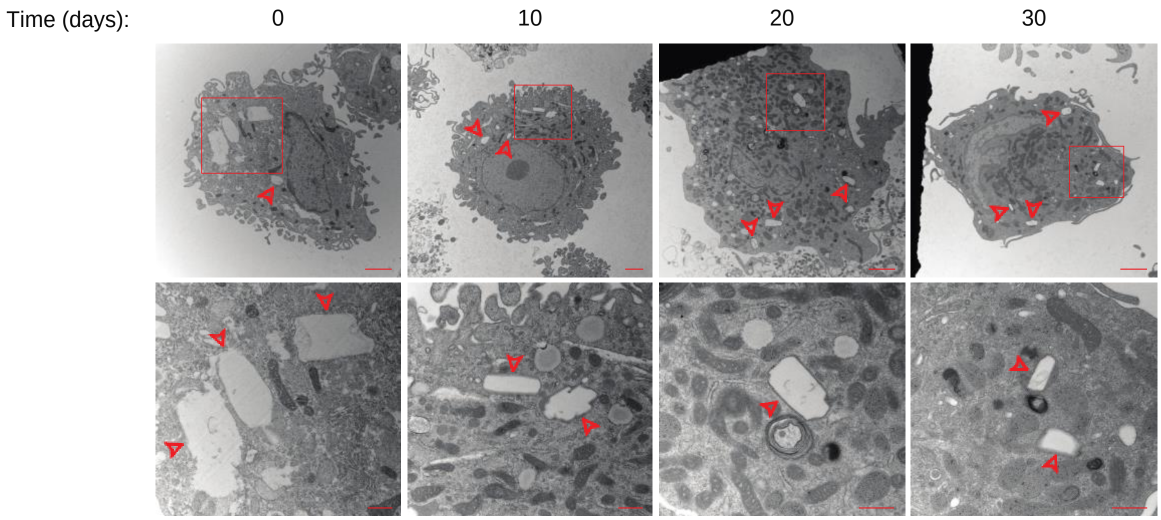

Supplementary Fig. 12. Transmission electron microscopy (TEM) of MDM treated with NM2DTG in vitro. TEM images of MDM after treatment with 25 μM NM2DTG for 8 h. Cells were collected immediately, 10, 20, or 30 days after treatment. N = 4 biological replicates with representative images shown. Red arrowheads indicate nanocrystals retained in the cytoplasm. Scale bars - 2 μm top, 500 nm bottom.

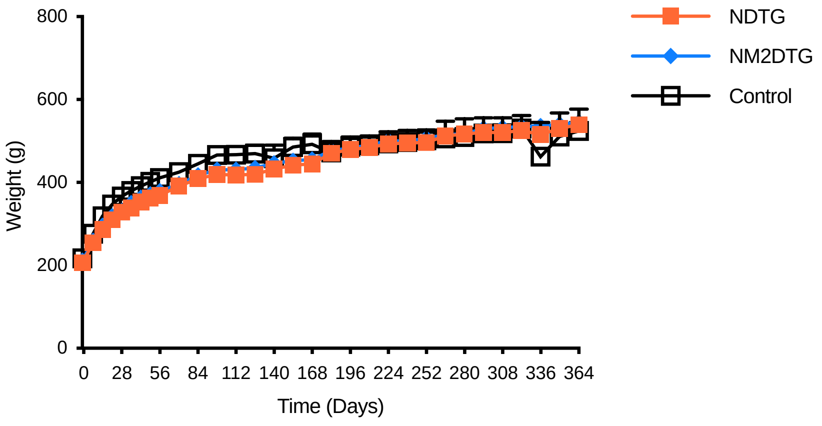

Supplementary Fig. 13. Toxicity of NM2DTG in male SD rats. Weight of male SD rats administered a single IM dose of NDTG (orange) or NM2DTG (blue) at 45 mg DTG-eq./kg in the caudal thigh up to day 364. Results are expressed as mean ± SEM. Study was initiated with N = 13 animals per treatment group (13 per group up to day 57, 9-10 per group up to day 175, and 5-6 per group up to day 364) and N = 4 animals for controls (black; 4 up to day 175 and 2 up to day 364). One animal was lost from the NDTG group on day 140 during the study period due to natural causes. Exact values for each time point provided in the source data. Source data are provided in the Figshare database under Digital Object Identifier (DOI) code 10.6084/m9.figshare.19027397.

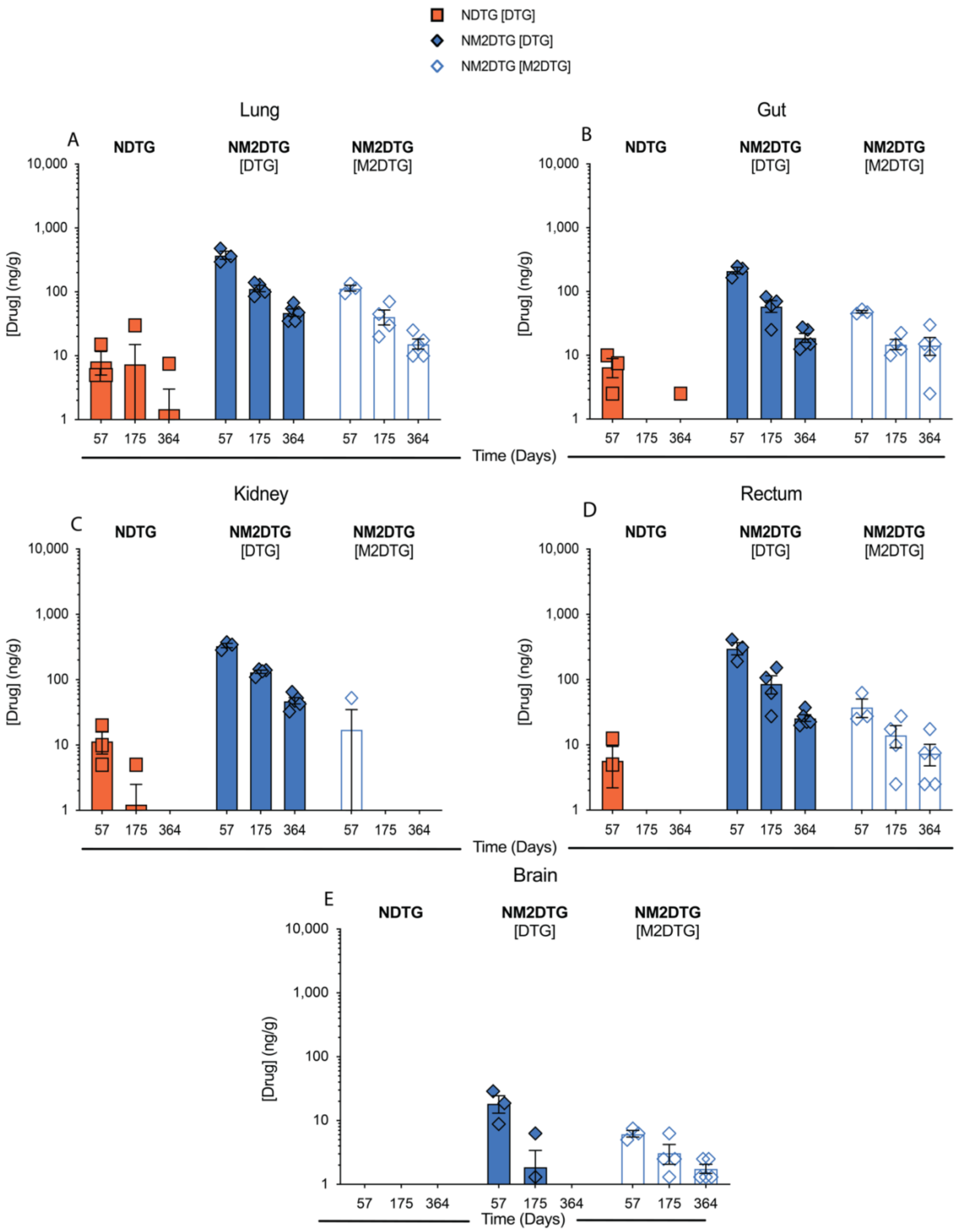

NDTG [DTG]
NM2DTG [DTG]
NM2DTG [M2DTG]
Lung
A
Gut
B
Kidney
C
Rectum
D
Brain
E
NDTG
NM2DTG [DTG]
NM2DTG [M2DTG]
[Drug] (ng/g)
10,000
1,000
100
10
1
57 175 364
Time (Days)

Supplementary Fig. 14. Biodistribution of NM2DTG in male SD rats. Male SD rats were administered with a single 45 mg DTG-eq./kg IM dose of NDTG (orange) or NM2DTG (blue) in the caudal thigh. Tissue biodistribution was assessed on days 57 (N = 3 animals per group), 175 (N = 4 animals per group), and 364 (N = 5 animals per group). Parent drug (DTG; solid blue diamonds) and prodrug (M2DTG; open blue diamonds) levels were determined in (**A**) lung, (**B**) gut, (**C**) kidney, (**D**) rectum, and (**E**) brain. All drug levels were quantified by UPLC-MS/MS. Results are expressed as mean ± SEM. Source data are provided in the Figshare database under Digital Object Identifier (DOI) code 10.6084/m9.figshare.19027397.

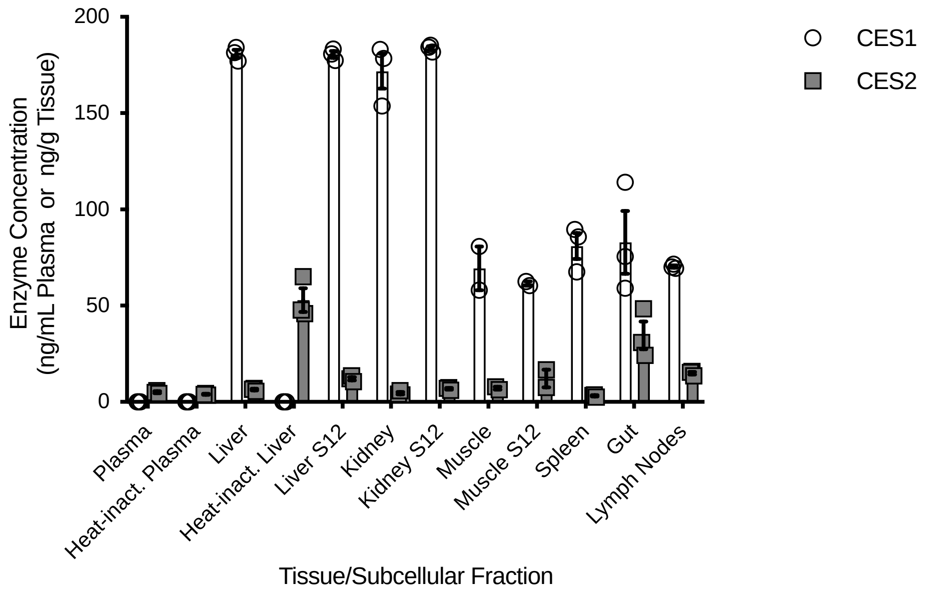

Supplementary Fig. 15. Carboxylesterase (CES) levels in rat tissue homogenates. Quantification of CES isoform, CES1 (open circles) and CES2 (grey squares), concentrations in various rat plasma, tissue homogenates, and subcellular fractions by ELISA. Results are expressed as mean ± SEM for N = 3 biological replicates. Source data are provided in the Figshare database under Digital Object Identifier (DOI) code 10.6084/m9.figshare.19027397.

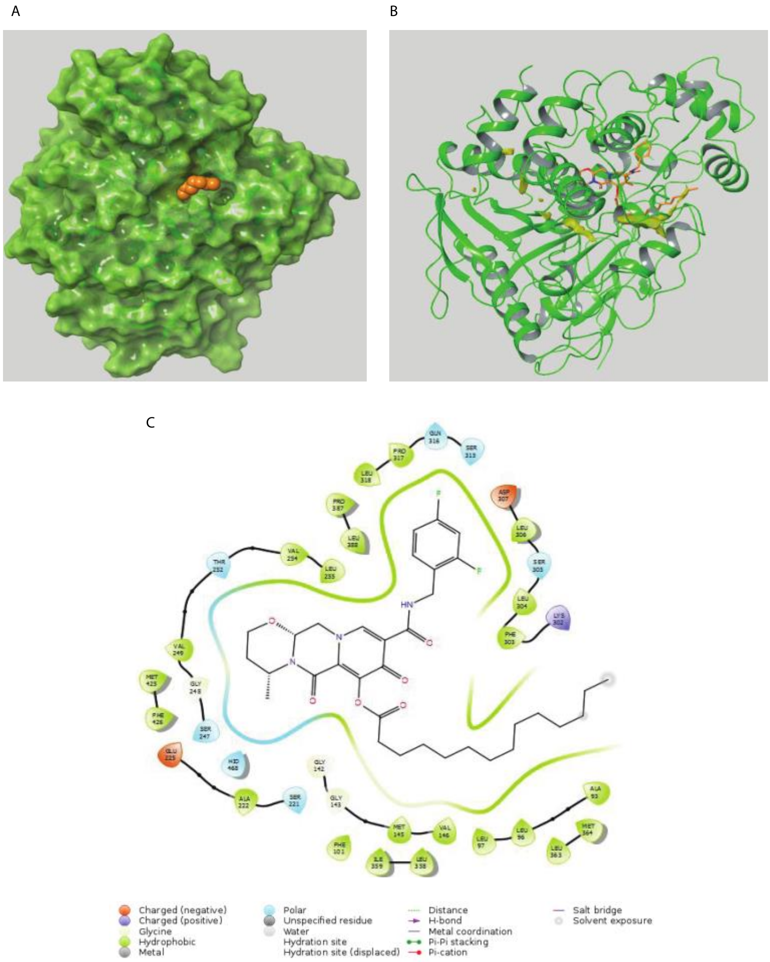
A
B
C
Charged (negative)
Charged (positive)
Glycine
Hydrophobic
Metal
Polar
Unspecified residue
Water
Hydration site
Hydration site (displaced)
Distance
H-bond
Metal coordination
Pi-Pi stacking
Pi-cation
Salt bridge
Solvent exposure

Supplementary Fig. 16. Molecular docking. (A) Three-dimensional crystal structures of carboxylesterase-1 (CES1; PDB ID: 1YA8; PDB DOI: 10.2210/pdb1YA8/pdb) protein were retrieved from the Research Collaboratory for Structural Bioinformatics Protein Data Bank (RCBS PDB) database). (B) Docking solution of MDTG docked to CES1 (1YA8); MDTG is orange, the hydrophobic patch is yellow, and the protein ribbon is green. (C) Two-dimensional ligand interaction maps of CES1 with MDTG. The interaction of M2DTG with the enzyme could not be mapped due to meager affinity.

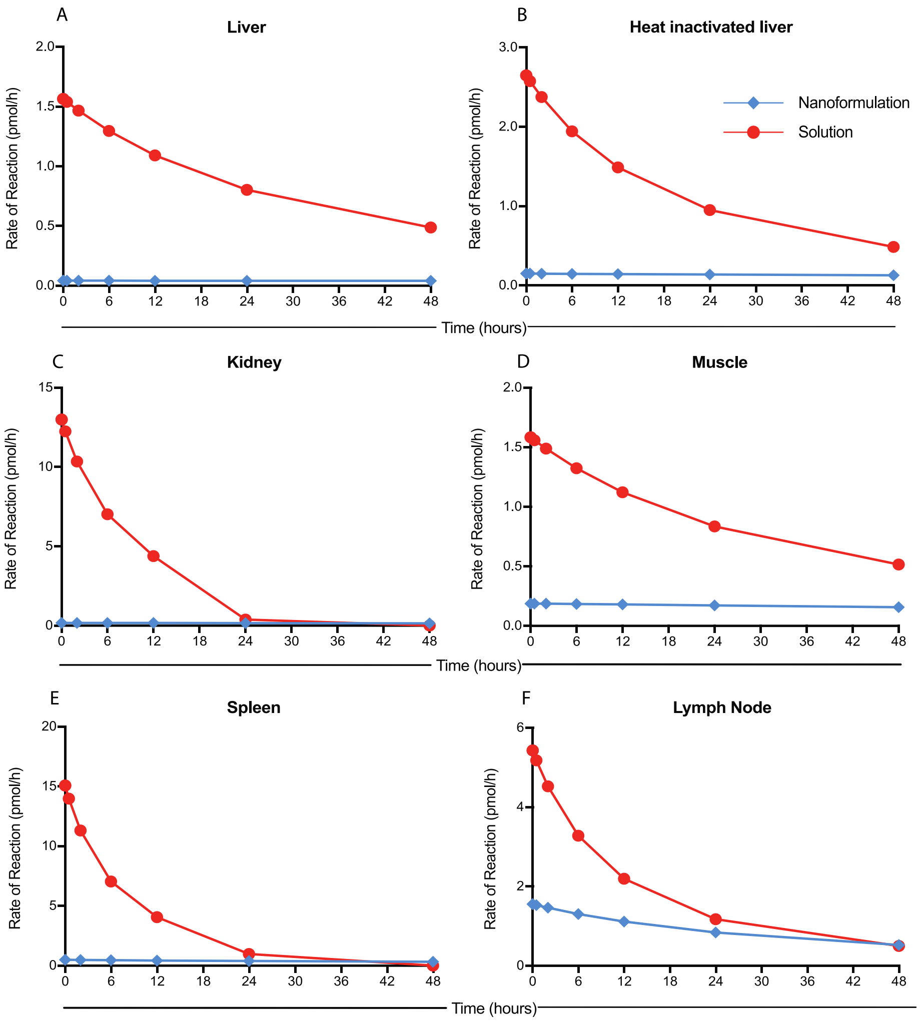
A
Liver
B
Heat inactivated liver
Nanoformulation
Solution
C
Kidney
D
Muscle
E
Spleen
F
Lymph Node
Rate of Reaction (pmol/h)
Time (hours)
0
6
12
18
24
30
36
42
48

Supplementary Fig. 17. Reaction rate kinetics of the cleavage of M2DTG and NM2DTG in rat tissue homogenates. Rate of reaction for cleavage of M2DTG solution (dissolved in 1% (v/v) methanol; red) and NM2DTG nanoformulation (blue) in (A) liver, (B) heat-inactivated liver, (C) kidney, (D) muscle, (E) spleen, and (F) lymph node. Results are calculated as the mean for N = 3 independent replicates/time point (N = 2 for lymph node due to limited sample). Source data are provided in the Figshare database under Digital Object Identifier (DOI) code 10.6084/m9.figshare.19027397.

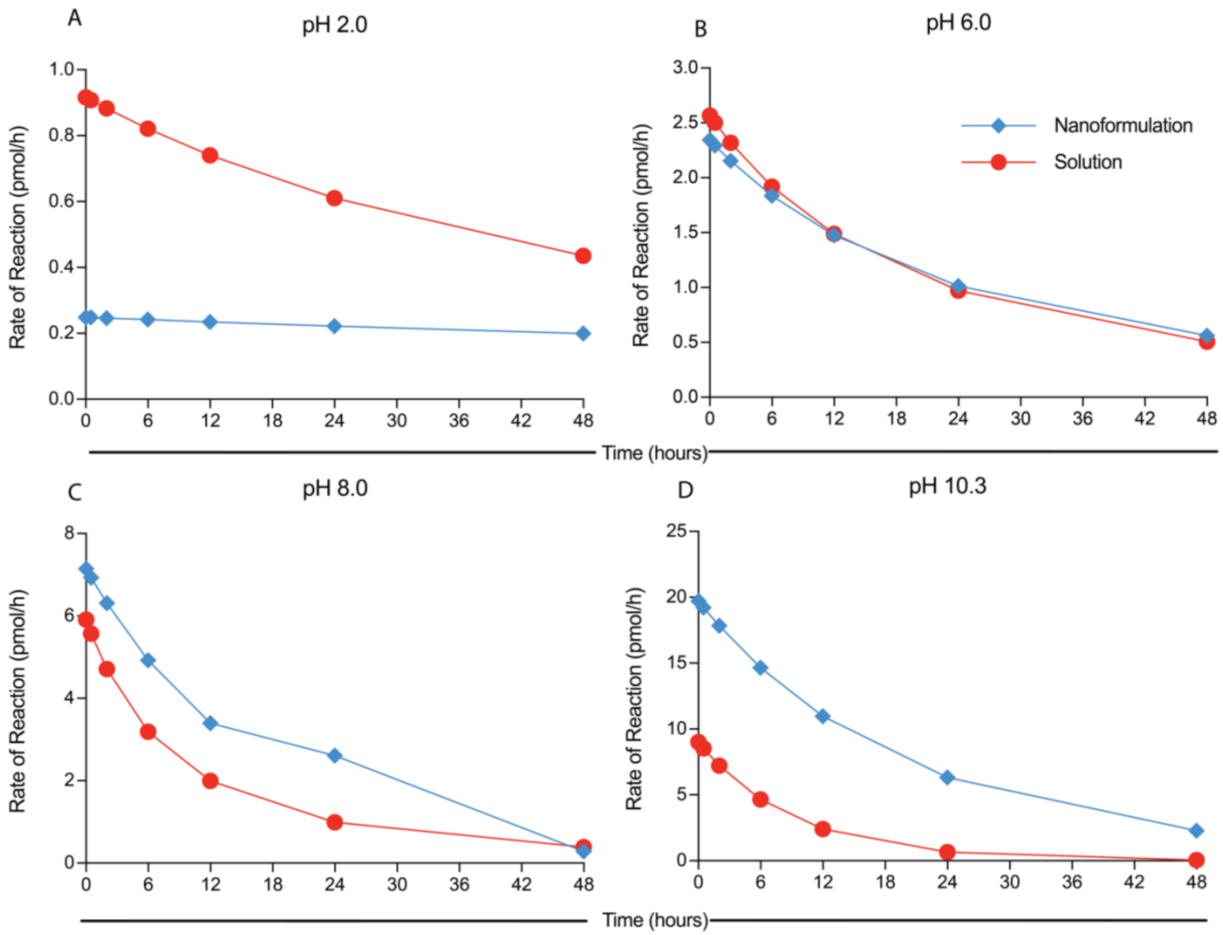
A
pH 2.0
B
pH 6.0
C
pH 8.0
D
pH 10.3
Rate of Reaction (pmol/h)
Time (hours)
Nanoformulation
Solution
0
6
12
18
24
30
36
42
48

Supplementary Fig. 18. Reaction rate kinetics of the hydrolysis of M2DTG and NM2DTG in various pH buffers. Rate of reaction for hydrolysis of M2DTG solution (dissolved in 1% (v/v) methanol; red) and NM2DTG nanoformulation (blue) in buffers of (A) pH 2.0, (B) pH 6.0, (C) pH 8.0, and (D) pH 10.3. Results are calculated as the mean for N = 3 independent replicates/time point. Source data are provided in the Figshare database under Digital Object Identifier (DOI) code 10.6084/m9.figshare.19027397.

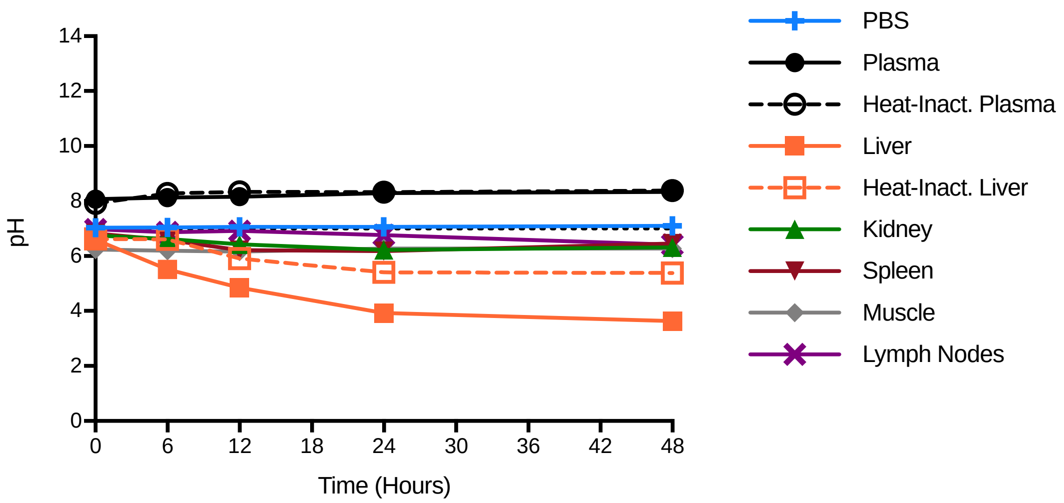

Supplementary Fig. 19. Changes in rat plasma and tissue homogenate pH over time. The pH of rat plasma and various tissue homogenates was measured after preparation at 0, 6, 12, 24, and 48 h with incubation at 37°C and mechanical agitation. Tissues were homogenized in 5 volumes of ice-cold PBS using a TissueLyser. Results are expressed as the mean for N = 3 independent replicates. Source data are provided in the Figshare database under Digital Object Identifier (DOI) code 10.6084/m9.figshare.19027397.

Supplementary Table 1. Rat serum chemistry analysis

Serum Chemistry				Day 3			Day 57						Day 175						Day 364					
	Control			NM2DTG			NDTG			NM2DTG			NDTG			NM2DTG			NDTG			NM2DTG		
	Mean	SEM	N	Mean	SEM	N	Mean	SEM	N	Mean	SEM	N	Mean	SEM	N	Mean	SEM	N	Mean	SEM	N	Mean	SEM	N
Albumin (g/dL)	4.2	0.3	4	4.0	0.0	3	4.8	0.2	3	5.2	0.1	3	4.6	0.2	4	4.0	0.4	4	4.6	0.3	5	4.2	0.3	6
Alkaline phosphatase (U/L)	226	24	4	405*	29	3	146	21	3	105	12	3	282	33	4	194	30	4	199	27	5	169	23	6
Alanine aminotransferase (U/L)	58	6	4	73	6	3	32	2	3	29	2	3	61	5	4	77	7	4	72	9	5	65	3	6
Amylase (U/L)	952	23	4	724*	21	3	792	23	3	688**	41	3	897	16	4	853	23	4	786	16	5	788	27	6
Total bilirubin (mg/dL)	0.3	0.0	4	0.2	0.0	3	0.2	0.0	3	0.2	0.0	3	0.2	0.0	4	0.3	0.0	4	0.3	0.0	5	0.3	0.0	6
Blood Urea Nitrogen (mg/dL)	22	1	4	18	1	3	23	1	3	26	1	3	22	1	4	27	9	4	19	0	5	18	1	6
Calcium (mg/dL)	11.7	0.1	4	12.3	0.1	3	9.3	0.4	3	10.0	0.4	3	11.8	0.2	4	11.6	0.3	4	11.6	0.2	5	11.8	0.2	6
Phosphorus (mg/dL)	6.8	0.3	4	11.8**	0.2	3	6.7	0.1	3	6.6	0.8	3	7.6	0.3	4	6.9	0.1	4	6.5	0.4	5	6.3	0.4	6
Creatinine (mg/dL)	0.4	0.1	4	0.4	0.1	3	0.3	0.1	3	0.6	0.0	3	0.4	0.1	4	0.4	0.1	4	0.4	0.0	5	0.4	0.1	6
Glucose (mg/dL)	145	10	4	185	6	3	162	16	3	155	84	3	166	15	4	196	21	4	127	9	5	159	17	6
Sodium (mmol/L)	145	3	4	144	1	3	141	2	3	143	1	3	143	1	4	143	1	4	133	1	5	132	1	6
Potassium (mmol/L)	8.0	0.3	4	6.0*	0.2	3	6.6	0.6	3	6.8	0.5	3	7.9	0.2	4	7.1	0.3	4	7.2	0.3	5	6.1*	0.2	6
Total protein (g/dL)	6.9	0.2	4	5.8	0.1	3	6.5	0.3	3	6.8	0.1	3	7.0	0.2	4	6.8	0.2	4	7.0	0.1	5	7.0	0.1	6
Globulin (g/dL)	2.7	0.2	4	1.9	0.1	3	1.8	0.0	3	1.7	0.0	3	2.4	0.1	4	2.8	0.4	4	2.5	0.2	5	2.9	0.2	6

Two-way ANOVA with Geisser-Greenhouse correction and Dunnett's multiple comparisons test (Control vs. Treatment) $*P < 0.05$, $**P < 0.01$. Control vs. NM2DTG, Day 3; Alkaline Phosphatase – $P = 0.0388$, Amylase – $P = 0.0148$, Phosphorous – $P = 0.0044$, Potassium – $P = 0.0432$. Control vs. NM2DTG, Day 57; Amylase – $P = 0.0100$. Control vs. NM2DTG, Day 364; Potassium – $P = 0.0407$. Source data are provided as a Source Data file.

Supplementary Table 2. Mouse pharmacokinetic profiles

PK Parameters	NDTG	NMDTG	NM2DTG	NM3DTG	NM4DTG
λ_z (1/day)	0.1991	0.0177	0.0041	0.0167	0.0304
$t_{1/2}$ (day)	3.48	39.09	167.90	41.51	22.77
AUC_{last} (day*ng/mL)	66561.10	53958.12	59294.17	2596.60	46454.75
$AUC_{0\text{-}inf}$ (day*ng/mL)	66562.71	54012.26	76152.92	4183.62	46469.20
AUC % Extrapolation	0.0024	0.1002	22.14	37.93	0.0311
V_z/F (L/kg)	3.40	46.99	143.13	644.16	31.81
CL/F (L/day/kg)	0.68	0.83	0.59	10.76	0.97
$MRT_{0\text{-}inf}$	7.31	34.57	248.60	54.85	52.35

Supplementary Table 3. Rat pharmacokinetic profiles

PK Parameters	NDTG	NM2DTG
λ_z (1/day)	0.1529	0.0064
$t_{1/2}$ (day)	4.53	108.76
AUC_{last} (day*ng/mL)	90740.00	78012.16
$AUC_{0\text{-}inf}$ (day*ng/mL)	90746.55	87112.41
AUC % Extrapolation	0.0072	10.45
V_z/F (L/kg)	3.24	82.72
CL/F (L/day/kg)	0.50	0.53
$MRT_{0\text{-}inf}$	5.74	147.36

Supplementary Table 4. Rhesus macaque pharmacokinetic profiles (Dose 1; Day 0-217)

PK Parameters	T006	T017	R036
Rsq adjusted	0.82	0.73	0.73
λ_z (1/day)	0.0088	0.0074	0.0080
$t_{1/2}$ (day)	78.49	93.82	86.32
AUC_{last} (day*ng/mL)	22379.87	23474.00	28804.68
$AUC_{0\text{-}inf}$ (day*ng/mL)	26909.12	29429.79	35280.53
AUC % Extrapolation	16.83	20.24	18.36
V_z/F (L/kg)	189.36	206.97	158.84
CL/F (L/day/kg)	1.67	1.53	1.28
$MRT_{0\text{-}inf}$	116.02	126.68	117.47

Supplementary Table 5. Rhesus macaque pharmacokinetic profiles (Dose 2; Day 217-terminal)

PK Parameters	T006	T017	R036
Rsq adjusted	0.97	0.86	0.94
λ_z (1/day)	0.0091	0.0068	0.0106
$t_{1/2}$ (day)	76.01	101.82	65.20
AUC_{last} (day*ng/mL)	24909.05	27322.57	31610.31
$AUC_{0\text{-}inf}$ (day*ng/mL)	29734.03	35548.98	37066.38
AUC % Extrapolation	16.23	23.14	14.72
V_z/F (L/kg)	165.96	185.95	114.20
CL/F (L/day/kg)	1.51	1.27	1.21
$MRT_{0\text{-}inf}$	102.26	141.76	60.20
