# Supplementary material for: Transformation of dolutegravir into an ultra-long-acting parenteral prodrug formulation
Source: Nat Commun. 2022 Jun 9;13:3226. doi: 10.1038/s41467-022-30902-7 (PMC9184486; doi:10.1038/s41467-022-30902-7)
Supplement: Supplementary file 3 — Reporting Summary [file 41467_2022_30902_MOESM3_ESM.pdf]

nature portfolio

Corresponding author(s): Benson Edagwa, PhD and Howard E. Gendelman, MD

Last updated by author(s): May 9, 2022

Reporting Summary

Nature Portfolio wishes to improve the reproducibility of the work that we publish. This form provides structure for consistency and transparency in reporting. For further information on Nature Portfolio policies, see our Editorial Policies and the Editorial Policy Checklist.

Statistics

For all statistical analyses, confirm that the following items are present in the figure legend, table legend, main text, or Methods section.

n/a	Confirmed	
☐	☒	The exact sample size (*n*) for each experimental group/condition, given as a discrete number and unit of measurement
☐	☒	A statement on whether measurements were taken from distinct samples or whether the same sample was measured repeatedly
☐	☒	The statistical test(s) used AND whether they are one- or two-sided<br>*Only common tests should be described solely by name; describe more complex techniques in the Methods section.*
☒	☐	A description of all covariates tested
☐	☒	A description of any assumptions or corrections, such as tests of normality and adjustment for multiple comparisons
☐	☒	A full description of the statistical parameters including central tendency (e.g. means) or other basic estimates (e.g. regression coefficient) AND variation (e.g. standard deviation) or associated estimates of uncertainty (e.g. confidence intervals)
☐	☒	For null hypothesis testing, the test statistic (e.g. *F*, *t*, *r*) with confidence intervals, effect sizes, degrees of freedom and *P* value noted<br>*Give P values as exact values whenever suitable.*
☒	☐	For Bayesian analysis, information on the choice of priors and Markov chain Monte Carlo settings
☒	☐	For hierarchical and complex designs, identification of the appropriate level for tests and full reporting of outcomes
☒	☐	Estimates of effect sizes (e.g. Cohen's *d*, Pearson's *r*), indicating how they were calculated

Our web collection on statistics for biologists contains articles on many of the points above.

Software and code

Policy information about availability of computer code

Data collection	Empower 3 software (Waters, Milford, MA); MassLynx V4.2 software (Waters, Milford, MA); TRIOS software V5.1.1 (Waters/TA Instruments, New Castle, DE); SoftMax Pro 6.2 software (Sunnyvale, CA); Maestro Suite 2020-2 software (Schrodinger, New York City, NY, USA)
Data analysis	Empower 3 software (Waters, Milford, MA); MassLynx V4.2 software (Waters, Milford, MA); Microsoft Excel V16.49 (Redmond, WA); GraphPad Prism V9.3.0 (San Diego, CA); Non-compartmental PK analysis for plasma drug levels was performed using Phoenix WinNonlin-8.3.3.33 software (Certara, Princeton, NJ)

For manuscripts utilizing custom algorithms or software that are central to the research but not yet described in published literature, software must be made available to editors and reviewers. We strongly encourage code deposition in a community repository (e.g. GitHub). See the Nature Portfolio guidelines for submitting code & software for further information.

Data

Policy information about availability of data

All manuscripts must include a data availability statement. This statement should provide the following information, where applicable:

- Accession codes, unique identifiers, or web links for publicly available datasets
- A description of any restrictions on data availability
- For clinical datasets or third party data, please ensure that the statement adheres to our policy

The authors declare that the data supporting the findings of this study are available within the paper and its supplementary information files. Source data are provided with this paper.
DOIs: 10.6084/m9.figshare.19026452 (Fig. 1), 10.6084/m9.figshare.19026983 (Fig. 2), 10.6084/m9.figshare.19027175 (Fig. 3), 10.6084/m9.figshare.19027229 (Fig. 4), 10.6084/m9.figshare.19027253 (Fig. 5), 10.6084/m9.figshare.19362500 (Fig. 6), 10.6084/m9.figshare.19027397 (supplementary materials).

The three-dimensional crystal structures of CES1 (PDB ID: 1YA8; PDB DOI: 10.2210/pdb1YA8/pdb) protein were retrieved from the Research Collaboratory for Structural Bioinformatics Protein Data Bank (RCBS PDB) database.

Field-specific reporting

Please select the one below that is the best fit for your research. If you are not sure, read the appropriate sections before making your selection.

- [x] Life sciences
- [ ] Behavioural & social sciences
- [ ] Ecological, evolutionary & environmental sciences

For a reference copy of the document with all sections, see nature.com/documents/nr-reporting-summary-flat.pdf

Life sciences study design

All studies must disclose on these points even when the disclosure is negative.

Sample size	Sample sizes chosen were sufficient to determine significance in all assays, with reproducible statistically significant differences between experimental conditions. For comparing two groups for PK analysis, six animals/group will provide 80% power at the 0.05 level of significance to detect a difference of 2.0 standard deviations using a t-test. For animal studies, sample sizes were determined in order to provide statistical power while also meeting cost and ethical criteria for animal use.
Data exclusions	Exclusion criteria was predetermined. Extreme outliers beyond the 99% confidence interval of the mean and 3-fold greater than the SEM were excluded. Excluded values are highlighted in blue with an asterisks in the raw data files.
Replication	All attempts to reproduce the experimental findings were successful. For chemical synthesis, characterization, and formulation production, experiments were repeated independently a minimum of three times with similar results. For in vitro cellular assays, experiments were repeated independently a minimum three times with similar results. For prodrug hydrolysis studies, experiments were repeated independently two times with equivalent results. For animal studies, results of the year long study were validated in three different species (mice, rats and rhesus macaques). Mouse studies were conducted once with a starting N = 5 animals per group, rat experiments were conducted once with a starting N = 13 animals per group (subdivided into three different sacrifice time points where N = 3 for 2 months, N = 4 for 6 months, and N = 6 for 1 year), and rhesus macaque studies were conducted once with an N = 3 animals.
Randomization	For all studies, samples/cells/animals were randomly allocated into experimental groups at the beginning of each study.
Blinding	Due to limitations in personnel numbers, experience, and trainings/clearance investigators were not blinded in conducting experiments or sample collection; instead relying on an unbiased approach. However, attempts were made to generate unbiased data through blinded data collection/analysis. For example, separate investigators conducted sample collection and data collection/analysis for all animal experiments (drug level determination, CBC counts, serum chemistry analysis), providing support to the unbiased conduct of the data generated. For animal studies, animals were also randomized into treatment groups before study initiation. For pathological evaluation of histology sections of tissues, pathologist was blinded.

Reporting for specific materials, systems and methods

We require information from authors about some types of materials, experimental systems and methods used in many studies. Here, indicate whether each material, system or method listed is relevant to your study. If you are not sure if a list item applies to your research, read the appropriate section before selecting a response.

Materials & experimental systems

n/a	Involved in the study	
☐	☒	Antibodies
☐	☒	Eukaryotic cell lines
☒	☐	Palaeontology and archaeology
☐	☒	Animals and other organisms
☒	☐	Human research participants
☒	☐	Clinical data
☒	☐	Dual use research of concern

Methods

n/a	Involved in the study	
☒	☐	ChIP-seq
☒	☐	Flow cytometry
☒	☐	MRI-based neuroimaging

Antibodies

Antibodies used	Monoclonal mouse anti-human HIV-1p24 [Catlaog #: sc-65918; Clone: 05-001; Lot #: K0420; Santa Cruz Biotechnology (Original Manufacturer: Dako, then Agilent, then Santa Cruz Biotechnology), Dallas, TX)]. Polymer-based HRP-conjugated anti-mouse EnVision+ secondary [Catlaog #: K400111-2; Clone: K4001; Lot #: 10137956; Agilent Tehnologies (Original Manufacturer: Dako, then Agilent Technologies), Santa Clara, CA)].
Validation	HIV-1p24 antibody and secondary provide excellent specificity, high lot to lot consistency, and certified manufacturing facilities guarantee full quality control (per company website). Antibody and secondary were validated and reported in previous publications [Nkwe DO. et al. 2016, Gnanadhas DP. et al. 2017; Sillman, B. et al. 2018; Hilaire JR. et al. 2019; Soni D. et al 2019; Smith N. et al

2019; Kulkarni TA. et al. 2020].

Eukaryotic cell lines

Policy information about cell lines

Cell line source(s)	Human peripheral blood monocytes
Authentication	Human peripheral blood monocytes were isolated by leukapheresis from HIV-1/2 and hepatitis B seronegative donors and purified by centrifugal elutriation from the University of Nebraska Medical Center (UNMC) Elutriation and Cell Separation Core according to a UNMC Institutional Review Board exempt protocol with informed consent. Monocytes were cultured for the first seven days in macrophage colony stimulating factor enriched media (MCSF, 1000 U/ml) to facilitate cell vitality and differentiation into macrophages.
Mycoplasma contamination	Cells obtained from elutriation were negative for mycoplasma
Commonly misidentified lines (See ICLAC register)	N/A

Animals and other organisms

Policy information about studies involving animals; ARRIVE guidelines recommended for reporting animal research

Laboratory animals	A) Mice: BALB/cJ, male, 6-8 weeks<br>B) Rats: Sprague Dawley, male, 8 weeks<br>C) Rhesus Macaques: Macaca mulatta, female, 8-13 years
Wild animals	The study did not involve wild animals.
Field-collected samples	The study did not involve samples collected from the field.
Ethics oversight	All experimental protocols involving the use of laboratory animals were approved by the UNMC Institutional Animal Care and Use Committee (IACUC) ensuring the ethical care and use of laboratory animals in experimental research. All animal studies were performed in compliance with institutional policies and NIH guidelines for laboratory animal housing and care in American Animal Association and Laboratory Animal Care (AAALAC) accredited facilities.

Note that full information on the approval of the study protocol must also be provided in the manuscript.
